# Mr. Ring, on Bronchocele

**Published:** 1800-11

**Authors:** John Ring

**Affiliations:** New Street, Hanover Square


### *To the Editors of the Medical and Phyfical Journal.*

Gentlemen,

HAVING frequently met with cafes of Bronchocele, which were given up as incurable by thofe who had been attending them, but yielded without much difficulty to a fimple and fafe remedy, I herewith fend the formula, which I beg the favour of you to infert in your Journal.

I am well aware that to medical practitioners in general, burnt fponge is known to be the bafis of the Coventry remedy, and to have been often given for this complaint; but, neverthelefs, facts warrant me to affirm, that either the cure of the difeafe is not in general well underftood, or that the means are not employed with due regularity and attention.

I have many times known external ftimulants, and mercurials, tried in vain; and fometimes the fpongia ufta itfelf inwardly given in other forms to no purpofe; yet in the following manner it has fucceeded.

℞. Spong. uft. ℥ij. pul. gum. Arab. ʒij. pul. cinnom. ʒfs. fyr. fimpl. q. s. ut fiant trochifci xxiv.

Care

Care muſt be taken that no more ſyrup be uſed than is abſolutely neceſſary to make the dry ingredients properly cohere; for which reaſon it muſt be added ſlowly, and the maſs muſt be beaten well. The lozenges are to be dried before the fire, on a plate that has been ſlightly oiled, to prevent them from ſticking; and muſt be kept in a bottle, or in a gallipot, tied over with ſkin.

One of them is to be taken twice or three times a day. I have known an inſtance where one was taken twice a day, for a great length of time, to no purpoſe; but when the number was increaſed to three, the good effect was ſoon evident.

Whether this medicine acts locally, and, if it acts locally, whether it is conveyed to the thyroid gland by means of abſorbents not hitherto diſcovered; or whether the thyroid gland is a mucous gland, and is ſtimulated to excretion by the action of this medicine on the neighbouring parts, I ſhall not pretend to determine. Suffice it to ſay, that I have cured a conſiderable number of perſons of the Bronchocele by this remedy, ſome of whom began to ſuffer much, and to be ſeriouſly alarmed, on account of the difficulty of deglutition and reſpiration, with which their complaints were at that time attended.

Burnt Sponge, it is well known, and has long been well known, is the moſt common remedy that is tried for the cure of this diſorder; but I have had frequent opportunities of knowing that it generally fails to effect a cure. This muſt proceed from the difference of the quantity given; or of the mode in which it is given. Some have adminiſtered it in the form of a ſoft bolus, to be diſſolved in the mouth. If its virtue depends, in any degree, upon its topical action, that muſt be very tranſient, unleſs the compoſition be dried.

Some years ago, I ſent a paper on this ſubject for publication to the Editor of a Medical Journal, who expreſſed a wiſh, that, previous to its publication, I would endeavour to aſcertain whether the ſpongia uſta really cures the diſeaſe in a ſhorter ſpace of time, when exhibited in this manner, than when ſwallowed at once.

I have made many attempts to decide this point; but, owing to the obſtinacy and impatience of the perſons who have laboured under the diſorder, and other circumſtances, I have hitherto been baffled in my endeavours. The frequent occurrence of the diſeaſe, and the frequent diſappointments that attend the ordinary modes which are employed for its removal, induce me to think that the publication of this formula may be uſeful, and ought not to be longer delayed.

In theſe troches the doſe of ſpongia uſta is a ſcruple. Some have recommended only that quantity, or perhaps only half a ſcruple, once a day; and that only during a particular period of of the moon. It is no wonder if fo fmall a quantity of the medicine fhould often fail to produce a cure.

Did not this mode of exhibiting the burnt fponge, in fome meafure, create difguft, and excite naufea, in many perfons of a delicate conftitution, fuch as is generally the lot of thofe who labour under this complaint, a much larger quantity of the medicine might be prefcribed with perfect fafety. The late Mr. Webb, of Half-Moon Street, informed me, that he cured a large fchirrous tumour of the abdomen, in a patient fixteen years old, by giving a drachm of it four times a day, till a pound was taken.

A kind of lozenge was prepared, and fold as a noftrum, many years ago, by a Frenchman of the name of Barbais; in which I had great reafon to believe burnt fponge was a principal ingredient. Thefe lozenges were fold at the enormous price of one guinea per pot; and each pot contained but a moderate quantity of the medicine.

I have feen a confiderable number of letters, from various parts of the kingdom, but particularly from Blandford, and that neighbourhood, defcribing, in the ftrongeft terms, the wonderful cures which had been performed by this remedy. Neverthelefs, owing to the indolence of the perfon who prepared them for Barbais, who was for many years a cripple, and on his own account, after the death of the proprietor, the fale was not fo great as it would otherwife have been.

Had the proprietor of this remedy practifed the refined arts of a modern quack, he might have emerged from obfcurity and have made his fortune; but he never folicited *peers of the realm* to certify more than was true, nor *a bifhop* or *a judge* to affure the world, that they were convinced of the fingular efficacy of *his noftrum*, from *their own experience.*

He did not open a warehoufe in New Bond Street for the fale of his medicine, nor fport his carriage or his country houfe; nor had he the impudence to affirm, as other empirics have done, that his patients had been given over by eminent phyficians, *who had never feen them*, *nor even heard of their illnefs.*

When I laft faw the perfon who prepared the medicine before alluded to, knowing that he had derived but little advantage from it of late years, owing to his being in an obfcure fituation—that of a fervant, and that he was of a very indolent difpofition, I offered to give him, what I confidered a reafonable confideration for his recipe, and if he would part with it on thofe terms, to make it public; but his demands were fo exorbitant, that I could not comply with them. He then lived with Captain Wade, and had lived many years with Colonel

Wade,

Wade, to whose father, Field Marshal Wade, the former proprietor had been a servant.

These particulars concerning a medicine which merits at least as high encomiums as any nostrum of the present day, will not, I hope, be deemed superfluous. I have great reason to believe, that burnt sponge was the basis of the composition, from the colour and taste of the lozenges; and from knowing that the person who prepared them, frequently purchased and calcined a considerable quantity of that article.

Having been very successful in the cure of the Bronchocele, I have long been anxious to communicate to medical practitioners in general, what I have embraced every opportunity of making known to individuals of the profession; and I sincerely hope, that by means of your widely circulated Journal, a knowledge of the great efficacy of this mild and safe medicine will be more generally diffused.

It is proper to add, that in a few cases, the same medicine, given in the form of pills, has cured the complaint; but I have not had it in my power to ascertain whether it will in general answer as well as when it is administered in the other form. I have the honour to be,

GENTLEMEN,

Your most obedient servant,

JOHN RING

*New Street, Hanover Square,*
*October* 9, 1800.

